# Impact of Two Water-Miscible Ionic Liquids on the Temperature-Dependent Self-Assembly of the (EO)_6_–(PO)_34_–(EO)_6_ Block Copolymer

**DOI:** 10.1021/acsomega.2c01166

**Published:** 2022-06-01

**Authors:** William T. Heller, Changwoo Do

**Affiliations:** Neutron Scattering Division, Oak Ridge National Laboratory, Oak Ridge, Tennessee 37831, United States

## Abstract

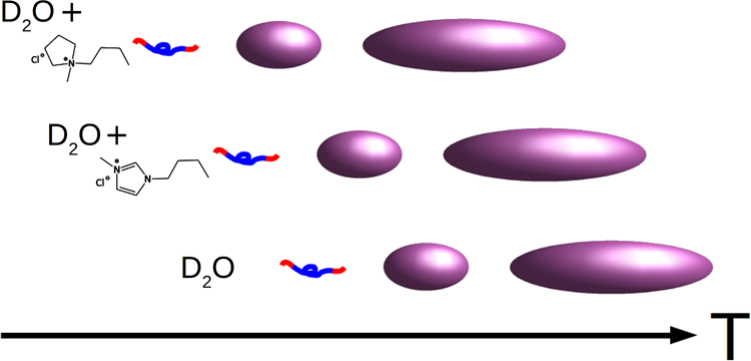

There are many studies on the self-assembly of triblock poly(ethylene oxide)–poly(propylene oxide)–poly(ethylene oxide) copolymers in aqueous solution. These polymers display a rich phase diagram as a function of block length, concentration, temperature, and additives. Here, we present a small-angle neutron scattering study of the impact of two water-miscible ionic liquids, 1-butyl-3-methylimidazolium chloride ([C_4_C_1_mim][Cl]) and 1-butyl-3-methylpyrrolidinium chloride ([C_4_C_1_pyrr][Cl]), on the temperature-dependent self-assembly of (EO)_6_–(PO)_34_–(EO)_6_, also known as L62 Pluronic, in aqueous solution. Both ionic liquids depress the temperatures of the various structural transitions that take place, but ([C_4_C_1_pyrr][Cl]) has a stronger effect. The structures that the triblock copolymer self-assembles into do not dramatically change nor do they significantly change the series of structures that the system transitions through as a function of temperature relative to the various transition temperatures.

## Introduction

The Pluronic triblock copolymers are composed of ethylene oxide (EO) and propylene oxide (PO) blocks in an (EO)_*m*_–(PO)_*n*_–(EO)_*m*_ arrangement. Variation of *m* and *n* produces a wide variety of physical properties due to the difference in hydrophobicity between EO and PO and the amphipathic nature of the copolymer’s structure, which makes the molecules surfactants. Pluronics self-assemble in solution, and aqueous solutions of the polymers have been extensively studied. The review by Alexandridis and Hatton^1^ gives an excellent overview of their behavior in solution. Pluronics continue to be studied due to their diversity, ready commercial availability, and potential for use in drug delivery. Readers are referred to Kabanov et al.,^2^ Chiappetta and Sosnick,^3^ and Batrakova and Kabanov^4^ for reviews about their use in drug delivery.

Even though Pluronics are not charged polymers, salts influence their interactions in water. The interaction of Pluronics and a variety of salts have been studied.^5−24^ The most studied salts are the alkali halides, but others have also been investigated. Broadly, salts impact both the critical micellization temperature (CMT) and the cloud point temperature, *T*_CP_. The effect generally follows the Hofmeister series,^25^ where there are examples of both “salting out” and “salting in”. K^+^ and Na^+^ decrease the CMT and *T*_CP_ more than Li^+^ or Mg^2+^. Similarly, the reduction in the CMT by F^–^ and Cl^–^ is greater than that by Br^–^. I^–^ and SCN^–^, which tend to cause “salting in”, increase the solubility of Pluronics.^10,14^ The salt-induced decrease in solubility correlates with an increase in the size of the micelles, which has been revealed by light scattering and small-angle neutron scattering.^7−9,14,19,20,22^ Systematic studies of the effect of the molecular weight of the polymer at a constant ratio of (EO) and (PO) do not show a strong correlation of the MW of the polymer with its response to salt.^18,20^

Ionic liquids (ILs), which are commonly defined as salts that are liquids below 100 °C, can be made with a wide variety of cations and anions.^26^ ILs continue to be studied because of their stability, recyclability, very low vapor pressure, and diverse physicochemical properties.^26−31^ Each cation and anion impart specific physical and chemical properties that give rise to the tunability that also makes ILs attractive. For example, ILs with imidazolium-based cations behave quite differently than those with a phosphonium-based cation.^26−31^ Similarly, varying the length of the alkyl groups of, for example, imidazolium-based cations alters the behavior of the resulting ILs.^32^ When one or both alkyl tails are sufficiently long, the ILs take on a surfactant character, and they are often referred to as surface-active ionic liquids (SAILs).^33^ In contrast, if the alkyl chains are short, the ILs can be fully water-miscible. It has been suggested that such ILs are hydrotropes,^34^ which can increase the solubility of hydrophobic compounds. Studies of ILs for biocatalytic applications resulted in them being ranked in the Hofmeister series.^35−41^

The L62 Pluronic has the structure (EO)_6_–(PO)_34_–(EO)_6_. The hydrophobic–lipophilic balance of the polymer is low, being 6–7,^1,42,43^ which places it among the hydrophobic Pluronics. Surface tension measurements of a 2 wt % solution indicated that the CMT is ∼28 °C.^44^ Various temperatures have been reported for the cloud point, although the manufacturer reports it to be 22–26 °C for a 10% aqueous solution on the product data sheet. For example, the CP of a 1 wt % solution was reported to be 32 °C,^1^ but another study revealed that it remains in an isotropic solution phase up to ∼60 °C for aqueous solutions containing up to ∼30 wt % Pluronic.^45^ Later studies determined that the behavior of the polymer was more complex. A concentration-dependent double cloud point was found for the polymer in aqueous solution.^46−48^ Small-angle neutron scattering (SANS) revealed that the polymer self-assembles in aqueous solution into a variety of structures as a function of temperature.^43^

L62, like many of the Pluronic polymers, has found a variety of applications where its detergent properties and relative safety and stability make it attractive. Here, the impact of two different water-miscible ILs, 1-butyl-3-methylimidazolium chloride ([C_4_C_1_mim][Cl]) and 1-butyl-3-methylpyrrolidinium chloride ([C_4_C_1_pyrr][Cl]), on the self-assembly of the Pluronic was studied as a function of IL concentration in water. The relative hydrophobicity of L62 and the identification of the two ILs as hydrotropes^34^ make determining how the ILs impact the self-assembly of L62 of interest. In particular, the ability to leverage the hydrotropic properties of the ILs to assist in incorporating hydrophobic compounds, such as additional active compounds when the polymer micelles are being used for cleaning applications, is of interest only if the ILs do not disrupt the structures formed by the polymer. Here, small-angle neutron scattering (SANS) was used to examine the temperature-dependent impact of these ILs at concentrations of up to 1.57 M in water on the self-assembly of L62 aqueous solutions containing 8.3 wt % polymer. The results provide new insight into the impact of water-miscible ILs on the self-assembly of the L62 Pluronic.

## Materials and Methods

### Materials and Sample Preparation

**Figure 1:**
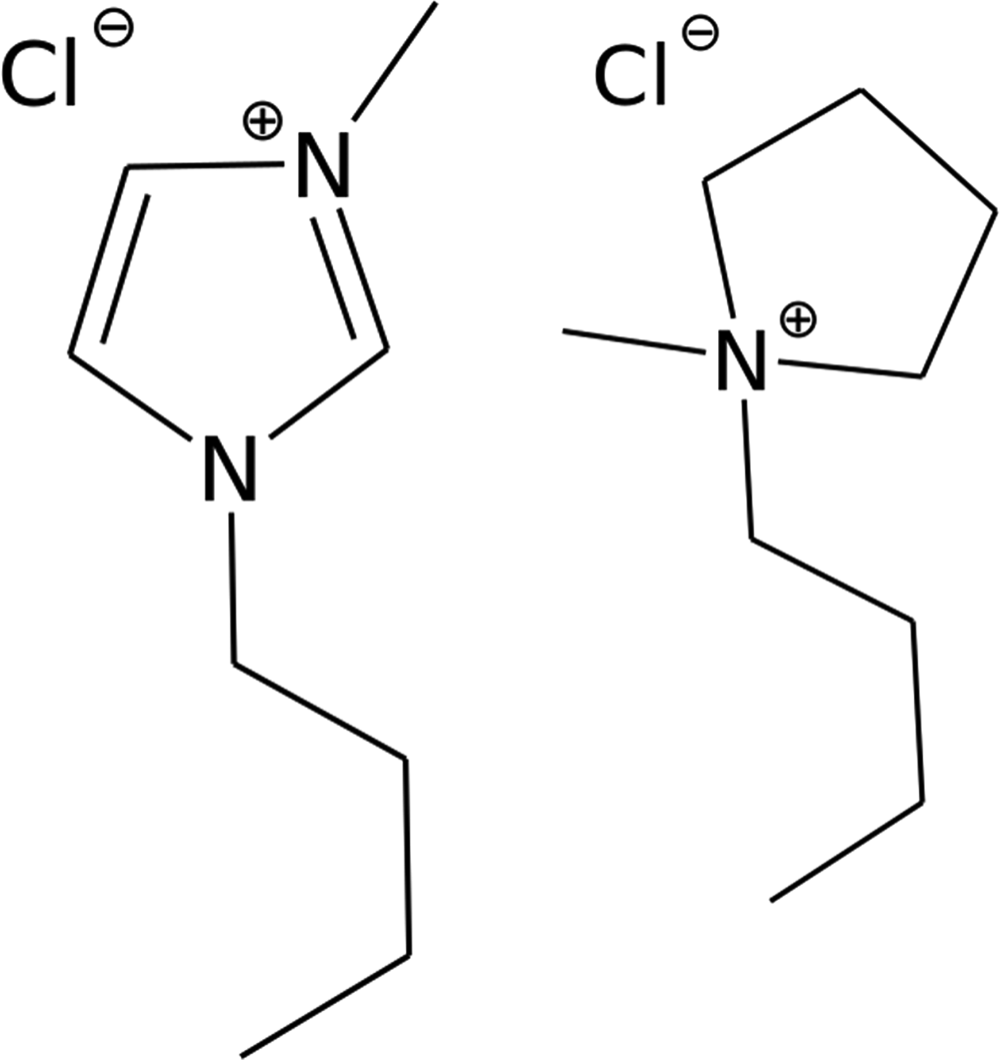
Chemical structures of [C_4_C_1_im][Cl] (left) and [C_4_C_1_pyrr][Cl] (right).

The (EO)_6_–(PO)_34_–(EO)_6_ (L62 Pluronic) block copolymer was a gift from BASF. D_2_O (99.8% D) was purchased from Cambridge Isotope Laboratories, Inc. (Tewksbury, MA). The ionic liquids, [C_4_C_1_im][Cl] and [C_4_C_1_pyrr][Cl], were purchased from Sigma-Aldrich (St. Louis, MO) and are shown in Figure 1. All materials were used without further purification. First, the mixtures of the ILs with D_2_O were made at IL concentrations of 0.10 M, 0.62 M (10 vol %), and 1.55 M (25 vol %) for [C_4_C_1_im][Cl] and 0.10 M, 0.63 M (10 vol %), and 1.57 M (25 vol %) for [C_4_C_1_pyrr][Cl]. Next, the (EO)_6_–(PO)_34_–(EO)_6_ was dissolved in D_2_O or the IL mixtures at a concentration of 8.3 wt % for all samples studied.

### Optical Absorbance Measurements

Optical absorbance measurements were performed to check the cloud points of the solutions studied. Measurements were made with a Shimadzu UV-2700 spectrophotometer, which is a dual-beam instrument that enabled the polymer-containing solutions to be measured against the corresponding solvents. The instrument has a TCC-240A temperature controller that is set manually. The difference in absorbance was monitored at a wavelength of 600 nm. Measurements were made at a series of temperatures from 10 to 55 °C in the pattern *T* = (10, 12, 15, 17, 20 °C, ...). The time between each temperature setting was 5 min. However, after the second temperature above the second cloud point, the time between absorbance readings was reduced to 2 min.

### Small-Angle Neutron Scattering (SANS)

SANS measurements were made using the EQ-SANS instrument at the Spallation Neutron Source of Oak Ridge National Laboratory.^49^ A single configuration was used for all measurements. A sample-to-detector distance of 4 m was employed with a minimum wavelength setting of 2.5 Å. The choppers ran at 30 Hz in the “frame-skipping” mode. As a result, the wavelength bands were 2.5–6.1 Å and 9.4–13.1 Å. This configuration provides a range of momentum transfers of 0.004 Å^–1^ < *q* < 0.45 Å^–1^, where *q* = 4π sin(θ)/λ, 2θ is the scattering angle, and λ is the wavelength. Samples were loaded into 1 mm path length cylindrical quartz cuvettes from Hellma (Plainview, NY). The original standard sample environment of the instrument^50^ was used to control the temperature to within ±1 °C by means of a water bath. Data reduction followed standard procedures.^51^ The data reduction included the subtraction of the appropriate solvent background.

### SANS Data Analysis

The neutron length densities (SLDs), ρ, of the materials in the samples were calculated from their chemical composition. The SLDs are presented in Table 1, along with the volumes of the various compounds in the samples. For the L62 triblock copolymer, *V*_L62_ = *V*_PPO_ + 2*V*_PEO_. *V*_PPO_ and *V*_PEO_ are also provided in Table 1. ρ_coil_ = (*V*_PPO_ ρ_PPO_ + 2*V*_PEO_ ρ_PEO_)/*V*_L62_, where ρ_PPO_ and ρ_PEO_ are the SLDs of the PPO and PEO, respectively, which are also presented in Table 1. The SLDs of the solvents were determined from the relative D_2_O and the IL content for each sample using the values in Table 1. The solvent SLDs are also presented in Table 1.

**Table 1:** Volumes and SLDs for the Chemicals and Solvents Used in the Samples Studied

chemical or solvent	volume (Å^3^)	ρ (10–6 Å^–2^)
D_2_O	30.0	6.38
[C_4_C_1_im][Cl]	97.4	2.61
[C_4_C_1_pyrr][Cl]	95.7	0.417
(PO)_34_	3286.0	0.343^43^
(EO)_6_	439.4	0.566^43^
(EO)_6_–(PO)_34_–(EO)_6_	4164.8	0.390^43^
0.10 M [C_4_C_1_im][Cl]		6.32
0.62 M [C_4_C_1_im][Cl]		6.00
1.55 M [C_4_C_1_im][Cl]		5.44
0.10 M [C_4_C_1_pyrr][Cl]		6.29
0.63 M [C_4_C_1_pyrr][Cl]		5.78
1.57 M [C_4_C_1_pyrr][Cl]		4.89

All data analysis was accomplished using the software *Sasview*.^52^ The information in Table 1 was used when the model being employed required SLD information. *Sasview*(52) made it possible to apply the β-correction for polydispersity to the structure factor^53^ to any model that included one during analysis. The SANS data were not fit over the entire *q*-range to avoid issues at the upper and lower ends of the *q*-range that result from inelastic scattering that occurs in the samples due to the presence of hydrogen and impacts the wavelength-dependent normalization of the time-of-flight data that is most evident at the high and low ends of the measured *q*-range.^54^ Instead, 0.008 Å^–1^ < *q* < 0.35 Å^–1^ was used during data analysis. In general, simpler models were used for data analysis, as was done previously,^43^ to avoid over-fitting the SANS data. The use of simpler models is also merited because of the very similar scattering length densities of [C_4_C_1_pyrr][Cl] and L62 that would make determining if the IL was integrated into structures that L62 self-assembles effectively impossible.

## Results

**Figure 2:**
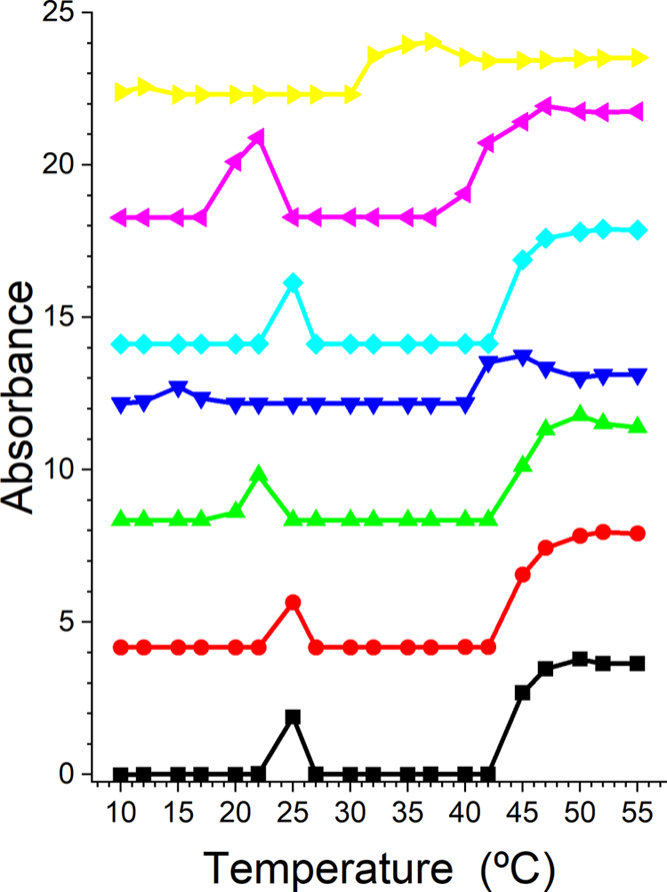
Absorbance as a function of temperature for L62 in D_2_O (black squares); 0.10 M (red circles), 0.62 M (green up triangles), and 1.55 M (blue down triangles) [C_4_C_1_im][Cl]; and 0.10 M (cyan diamonds), 0.63 M (purple left triangles), and 1.57 M (yellow right triangles) [C_4_C_1_pyrr][Cl]. The curves have been offset for clarity.

The absorbance data collected from the seven samples at the series of temperatures noted in the Materials and Methods section are shown in Figure 2. All samples show two temperature regions with high absorbance, which visual inspection confirmed are cloudy solutions. In the case of the solution without IL, both previously observed cloud point temperatures are observed at consistent temperatures.^46−48^ The upper CP also takes place at a temperature consistent with previous studies.^46−48^ When either [C_4_C_1_im][Cl] or [C_4_C_1_pyrr][Cl] is present at a concentration of 0.10 M, the CPs do not change significantly. However, the intermediate concentration of the ILs studied (0.62 M [C_4_C_1_im][Cl] and 0.63 M [C_4_C_1_pyrr][Cl]), the onset temperatures of the lower CPs decrease. The data also suggest that the temperature region of cloudiness is broader at this concentration of [C_4_C_1_pyrr][Cl]. The effect is stronger for [C_4_C_1_pyrr][Cl] than that for [C_4_C_1_im][Cl]. The upper CP is not significantly impacted by 0.62 M [C_4_C_1_im][Cl], but it decreases ∼5 °C when 0.63 M [C_4_C_1_pyrr][Cl] is present. The lower CP temperatures decrease, but the effect of [C_4_C_1_pyrr][Cl] is stronger. The impact of the highest concentrations of ILs studied on the CP temperatures is considerable, and the difference between the strength of their effects is amplified. Interestingly, while [C_4_C_1_im][Cl] only reduces the upper CP by ∼2–3 °C, the lower CP temperature has reduced by ∼10 °C. [C_4_C_1_pyrr][Cl] reduces the lower CP to near 10 °C, and the upper CP decreased to near 32 °C.

**Figure 3:**
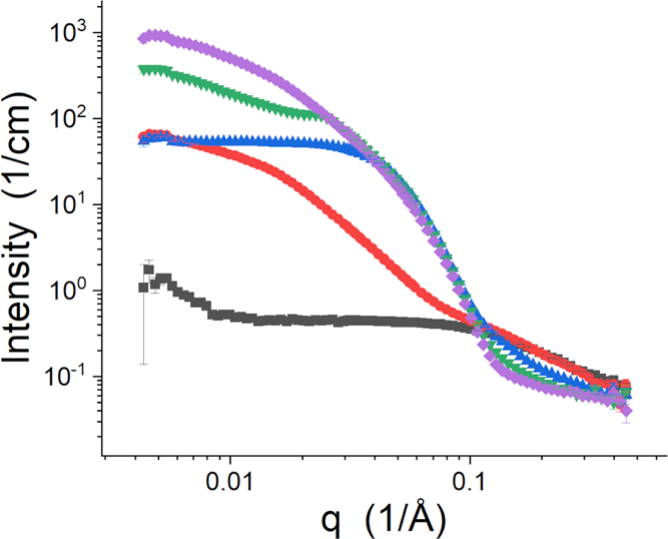
Temperature series of SANS data from L62 in D_2_O. The data were collected at 15 °C (black squares), 25 °C (red circles), 35 °C (blue up triangles), 45 °C (green down triangles), and 55 °C (purple diamonds).

The SANS data for the temperature series of L62 without salt are presented in Figure 3. At 15 °C, the Pluronic predominantly exists as free Gaussian chains in solution based on the power-law scattering for *q* > 0.15 Å^–1^. However, the data show some indication of a large structure, such as an aggregate, in the low-*q* region of the data based on the upturn for *q* < 0.02 Å^–1^. At 25 °C, the Pluronic assembles into what appear to be nonspecific, extended aggregates and free chains. This data set was measured at a temperature within the lower-temperature region identified as cloudy (Figure 2); so, the structure present at the length scales probed by SANS, being up to ∼1500 Å here, is part of the larger phase-separated structures that make the solution cloudy.^46−48^ The SANS data at 25 °C also share features of free Gaussian chains in the high-*q* region. The change in the high-*q* portion of the data between 25 and 35 °C suggests that the polymer chains are considerably more compact. Self-assembly progresses into larger structures as the temperature increases to 45 °C and then 55 °C. The two highest temperatures studied are above the second cloud point of the polymer in water^46−48^ and should be thought of as a local structure that is part of the phase-separated, cloudy solution, even though a previous SANS study modeled the structure at 55 °C as a large ellipsoid.^43^

**Figure 4:**
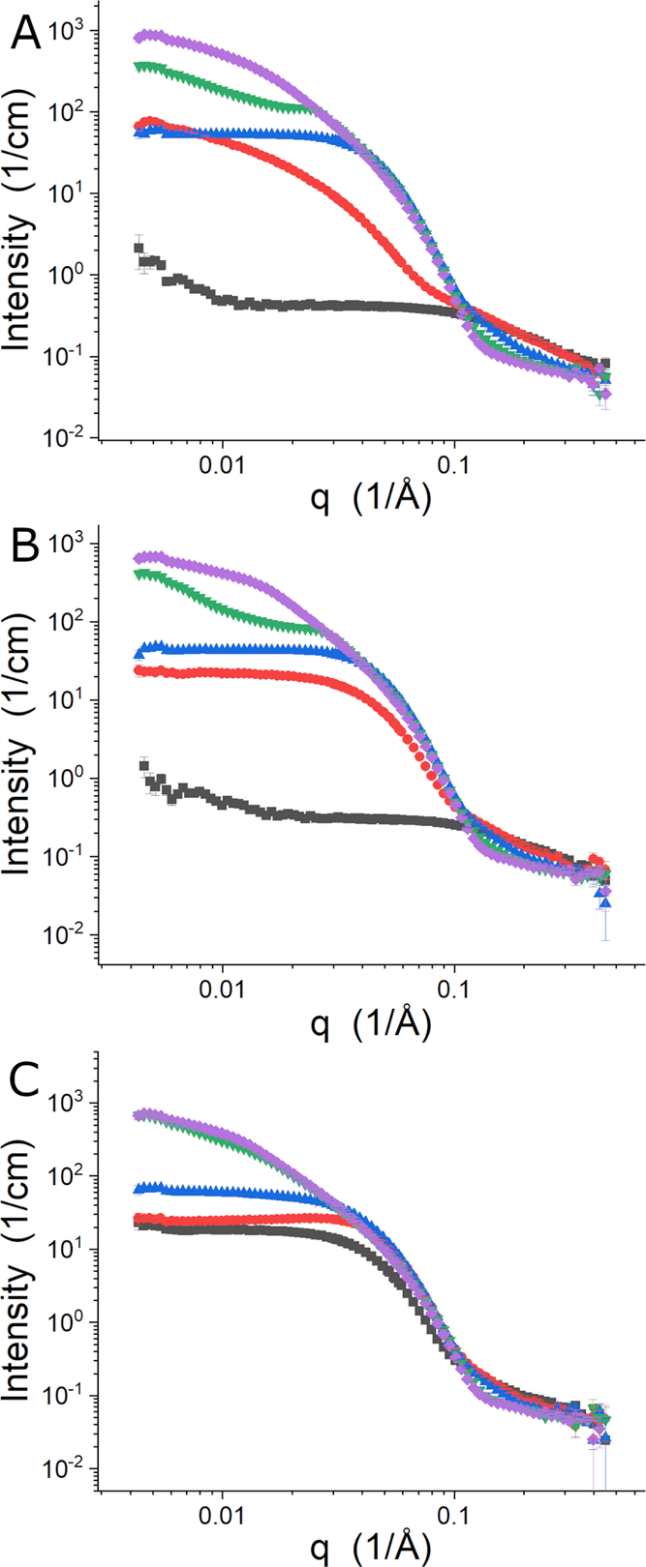
Temperature series of SANS data from L62 in D_2_O with 0.10 M (A), 0.62 M (B), and 1.55 M (C) [C_4_C_1_im][Cl]. The data were collected at 15 °C (black squares), 25 °C (red circles), 35 °C (blue up triangles), 45 °C (green down triangles), and 55 °C (purple diamonds).

SANS data collected for L62 in the presence of different concentrations of [C_4_C_1_im][Cl] are shown in Figure 4. The temperature series data collected for 0.10 M [C_4_C_1_im][Cl] shown in Figure 4A are very similar to that obtained without salt that is shown in Figure 3, indicating that the low concentration of the IL has produced little impact on the temperature-dependent self-assembly of the polymer. The influence of the IL is evident in Figure 4B (0.62 M) and Figure 4C (1.55 M). At 0.62 M [C_4_C_1_im][Cl], the Pluronic’s transition to micelles occurs at a lower temperature, and the nonspecific aggregates of Gaussian chains are not observed in the temperature series measured because the CP decreased. Instead, the micellar structure formed at 25 °C retains more scattering features of Gaussian coils at high-*q* than are present at 35 °C, which may be due to free unimers or the result of the EO blocks not adopting a more collapsed conformation in response to the IL. Based on the change in the strength of the signal and in the high-*q* data, the number density of the micelles increased between 25 and 35 °C due to a reduction in free unimers or the micelles became larger. The structures observed at 55 °C for the 0.10 M [C_4_C_1_im][Cl] sample are again observed, but the final structure shows indications that they are interacting based on the SANS data for *q* < 0.02 Å^–1^. An intermediate state again exists at 45 °C. No free unimers are evident in the 1.55 M [C_4_C_1_im][Cl] data sets, and the high-*q* data collected at all temperatures are more consistent with the 35 °C data from the 0.62 M [C_4_C_1_im][Cl] temperature series, which suggests that any population of free unimers is small. At 25 °C, there is a clear indication of a structure factor in the data that suggests that the strength of the interaction between the micelles changes between 15 and 35 °C while the structure transitions to larger micelles. The intermediate state observed at 45 °C for the two lower concentrations of [C_4_C_1_im][Cl] is not present. Instead, the large structure is found for the two highest temperatures studied, and they interact more at 55 °C based on the “knee” in the 0.01 Å^–1^ < *q* <0.02 Å^–1^ region.

**Figure 5:**
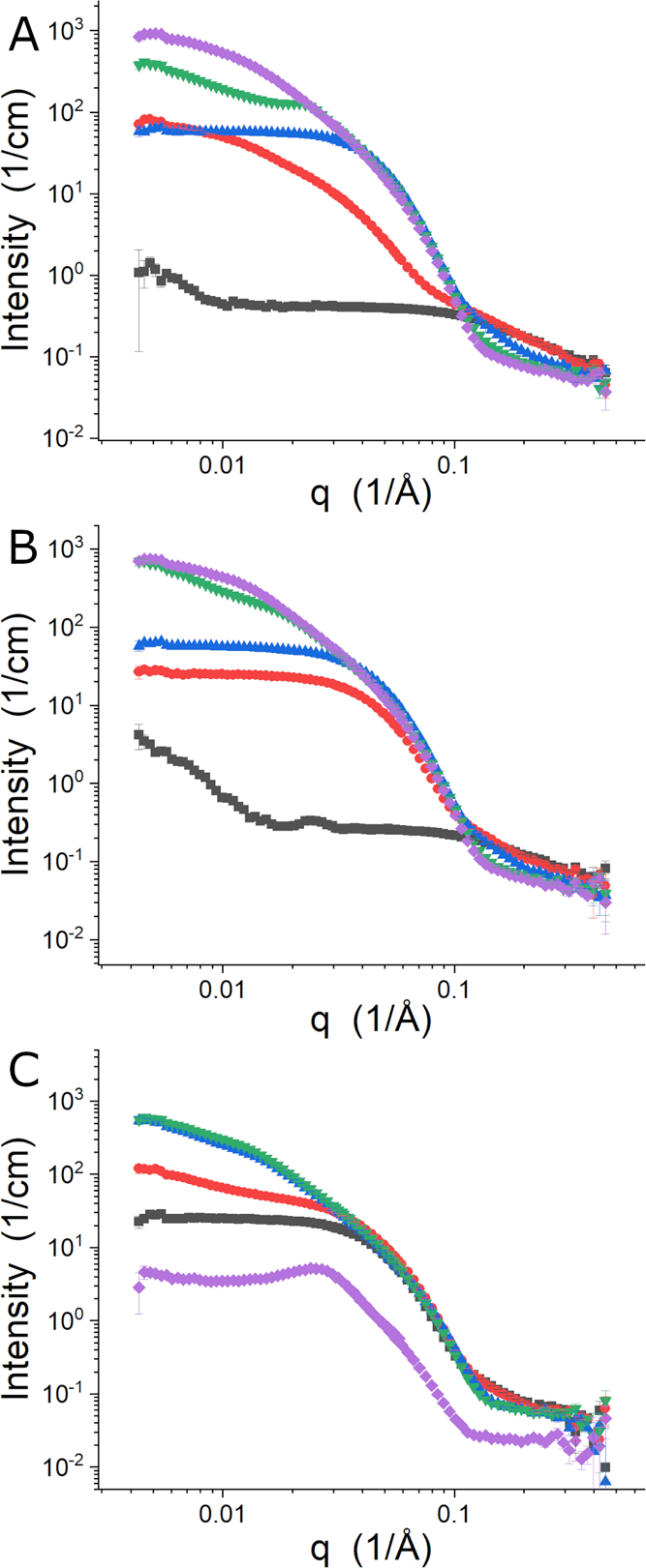
Temperature series of SANS data from L62 in D_2_O with 0.10 M (A), 0.63 M (B), and 1.57 M (C) [C_4_C_1_pyrr][Cl]. The data were collected at 15 °C (black squares), 25 °C (red circles), 35 °C (blue up triangles), 45 °C (green down triangles), and 55 °C (purple diamonds).

The SANS data collected for the temperature and [C_4_C_1_pyrr][Cl] concentration series are presented in Figure 5. When the IL concentration was 0.10 M, shown in Figure 5A, the Pluronic behaved much as it did when no salt or 0.10 M [C_4_C_1_im][Cl] was present. L62 unimers were free in solution at 15 °C, which gave way to nonspecific aggregates at 25 °C and then micelles at 35 °C. The self-assembled Pluronics then entered the intermediate transition state between micelles and the large structure at 45 °C, before forming the large structure at 55 °C. The first clear indications of a difference between the two ILs studied can be seen in the 0.63 M [C_4_C_1_pyrr][Cl] SANS data shown in Figure 5B. At 15 °C, there are stronger indications of a larger structure in the sample that manifest as the upturn at low-*q*, and there is what appears to be a diffraction peak in the data near *q* = 0.025 Å^–1^. The structures present at 25 and 35 °C are much like those found for the 0.63 [C_4_C_1_im][Cl] sample at the same temperature. Interestingly, the data at 45 °C are more like those collected at 55 °C, but the data suggest that the interaction between the structures is stronger at the higher temperature. [C_4_C_1_pyrr][Cl] at 1.57 M had the greatest impact on the structures formed by the Pluronic, as can be seen in Figure 5C. No free unimers were seen, like the 1.55 M [C_4_C_1_im][Cl] sample, but the temperatures for the various structures observed in the samples studied were lower in all cases. Importantly, a new phase was observed at 55 °C that has a characteristic correlation peak at *q* ∼ 0.025 Å^–1^.

**Figure 6:**
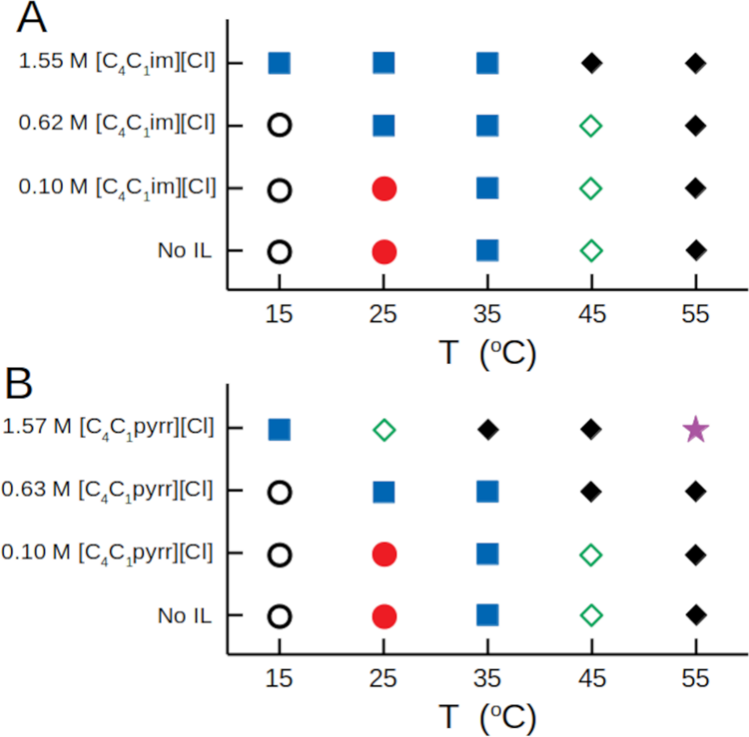
Structure categorization for L62 in the (A) [C_4_C_1_im][Cl] solutions and (B) [C_4_C_1_pyrr][Cl] solutions. The symbols correspond to predominantly Gaussian coils (open circles), nonspecific aggregates of Gaussian coils that are part of larger structures present in the phase-separated solutions (red circles), micelles (blue squares), a mixture of micelles and the large structure (green open diamonds), the large structure (black diamonds), and highly interacting structures (purple stars). These last two classifications are local structures that are part of the larger structures present in the phase-separated solutions.

The SANS data can be loosely categorized by the structure present. The categories chosen are unimers, nonspecific aggregates of unimers, micelles, the large structure, a mixture of micelles and the large structure, and highly interacting particles. These categorizations are presented in Figure 6. Increasing IL concentrations clearly promote the formation of larger aggregate structures of the L62 at lower temperatures. Further, [C_4_C_1_pyrr][Cl] promotes self-assembly to a greater extent than [C_4_C_1_im][Cl] regardless of the temperature.

**Figure 7:**
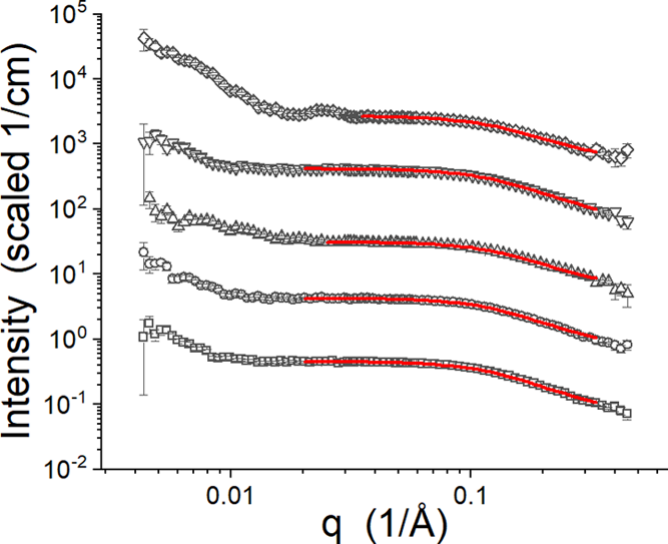
SANS data identified as Gaussian coils and the associated fit curves for no IL (squares), 0.10 M [C_4_C_1_im][Cl] (circles), 0.62 M [C_4_C_1_im][Cl] (up triangles), 0.10 M [C_4_C_1_pyrr][Cl] (down triangles), and 0.62 M [C_4_C_1_pyrr][Cl] (diamonds). The red curves are the fit lines. The data and fit curves have been offset for clarity.

To understand if the structures adopted by the Pluronic change in response to the ILs, select SANS data were analyzed. The SANS data were collected at specific temperatures rather than at specific temperatures relative to the CPs. The changes in the CPs made it possible that structures of interest were not present at one of the temperatures at which SANS data were collected. For example, the 1.57 M [C_4_C_1_pyrr][Cl] sample did not display data consistent with unimers at 15 °C. First, the data sets that showed indications of free unimers were fit using a Gaussian coil model^55^ with a hard-sphere structure factor^56^ to account for the high sample concentration. The fitting results are presented in Figure 7. Note that the q_min_ used for the fitting was high enough to reduce the impact of the larger structures that give rise to the features in the low-*q* region of the data, but it is reasonable to assume that the *R*_G_ values obtained from the fitting are larger than they might be if these large structures were not present. The *R*_G_ are presented in Table 2, and the fit curves are presented in Figure 7. The ILs caused the Pluronic to adopt a more compact conformation than when no IL was present. The volume fraction and effective radius used for the hard-sphere structure factor, also a free parameter, are presented in Table 2. The volume fractions are lower than might be expected based on the concentration of polymer used and they are small enough that they have little impact on the model intensities. The presence of the larger structure in the solutions that causes the upturns in the low-*q* region of the data in Figure 7 masks the impact of the polymer concentration on the SANS data.

**Table 2:** Results of Fitting SANS Data Identified as Gaussian Coils^a^

solvent	*R*_G_ (Å)	volume fraction	*R*_eff_ (Å)
no IL	13.5 ± 0.3	0.034 ± 0.003	19.1 ± 0.3
0.10 M [C_4_C_1_im][Cl]	11.7 ± 0.2	0.019 ± 0.002	23.0 ± 0.8
0.62 M [C_4_C_1_im][Cl]	11.4 ± 0.2	0.020 ± 0.001	22.3 ± 0.6
0.10 M [C_4_C_1_pyrr][Cl]	11.7 ± 0.1	0.018 ± 0.001	22.2 ± 0.5
0.63 M [C_4_C_1_pyrr][Cl]	11.6 ± 0.2	0.017 ± 0.001	21.7 ± 1.1

a The volume fraction and the effective radius, *R*_eff_, from the hard-sphere structure factor are also presented.

**Figure 8:**
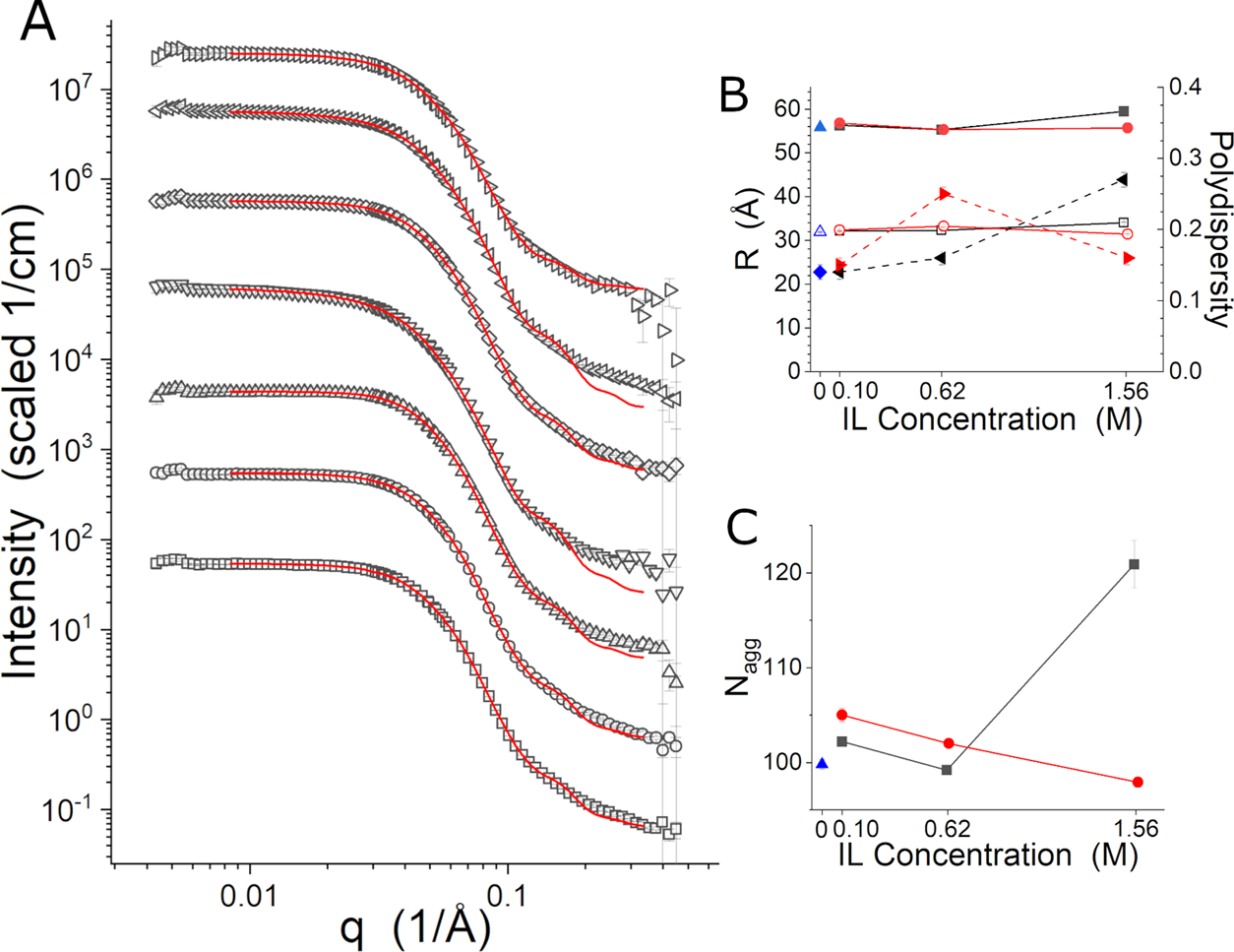
(A) SANS data identified as micelles and the associated fit curves for no IL at 35 °C (squares), 0.10 M [C_4_C_1_im][Cl] at 35 °C (circles), 0.62 M [C_4_C_1_im][Cl] at 35 °C (up triangles), 1.55 M [C_4_C_1_im][Cl] at 35 °C (down triangles), 0.10 M [C_4_C_1_pyrr][Cl] at 35 °C (diamonds), 0.63 M [C_4_C_1_pyrr][Cl] at 35 °C (left triangles), and 1.57 M [C_4_C_1_pyrr][Cl] at 15 °C (right triangles). The red curves are the fit lines. The data and fit curves have been offset for clarity. (B) *R*_equat_ and *R*_polar_ for no IL (blue solid and open up triangles), [C_4_C_1_im][Cl] (black solid and open squares), and [C_4_C_1_pyrr][Cl] (red solid and open circles), along with the polydispersity in *R*_equat_, Δ_equat_, for no IL (blue diamonds), [C_4_C_1_im][Cl] (black left triangles), and [C_4_C_1_pyrr][Cl] (red right triangles). (C) N_agg_ for no IL (blue up triangles), [C_4_C_1_im][Cl] (black squares), and [C_4_C_1_pyrr][Cl] (red circles).

The impact of the ILs on the micelles formed was also examined. The SANS data sets at the highest temperature identified as micelles in Figure 6 were selected for analysis because the number of free unimers appeared to be relatively low based on the appearance of the high-*q* data. The data sets analyzed were collected at 35 °C except for the 1.57 M [C_4_C_1_pyrr][Cl] sample, which was collected at 15 °C. These SANS data sets were fit using a polydisperse ellipsoid of rotation,^57^ and a hard-sphere structure factor^56^ was applied to account for the sample concentration used. The fits of the model to the data are shown in Figure 8A, while the polar (the axis of rotation) and equatorial radii, *R*_polar_ and *R*_equat_, respectively, and polydispersity in the equatorial radius Δ_equat_ that were found are presented in Figure 8B. The aggregation numbers, N_agg_, calculated from the ellipsoid volumes are presented in Figure 8C. The model profiles fit the data well, although there are deviations at high-*q*, which may be attributed to the surfaces of the micelles not being ideally ellipsoidal. These deviations may also be more evident because the SANS profiles have been corrected for the solvent scattering, rather than just the empty cell, which removes the incoherent background signal arising from the hydrogen in the solvent. Generally, the micelles are very similar in size. Aggregation numbers calculated from the volumes of the ellipsoids and the volume of the polymer (Table 1) range from 98 for the 1.57 M [C_4_C_1_pyrr][Cl] sample collected at 15 °C to 121 for the 1.55 [C_4_C_1_im][Cl] M sample collected at 35 °C, although most aggregation numbers are near 100, which is in reasonable agreement with a previous study of L62 studied at 1 wt % and in the presence of sodium xylene sulfonate.^48^ The 1.55 M [C_4_C_1_im][Cl] sample data set shows signs that the sample temperature is closer to the transition temperature of the next structural state. The dimensions of the ellipsoids for this sample at this temperature are consistently larger than the others. The polydispersity is also higher. In the case of the 1.57 M [C_4_C_1_pyrr][Cl] sample measured at the lower temperature of 15 °C, it can be inferred that the trend with concentration of this IL is the result of the sample being further below its transition to the next structural state than the 1.55 M [C_4_C_1_im][Cl] sample.

**Figure 9:**
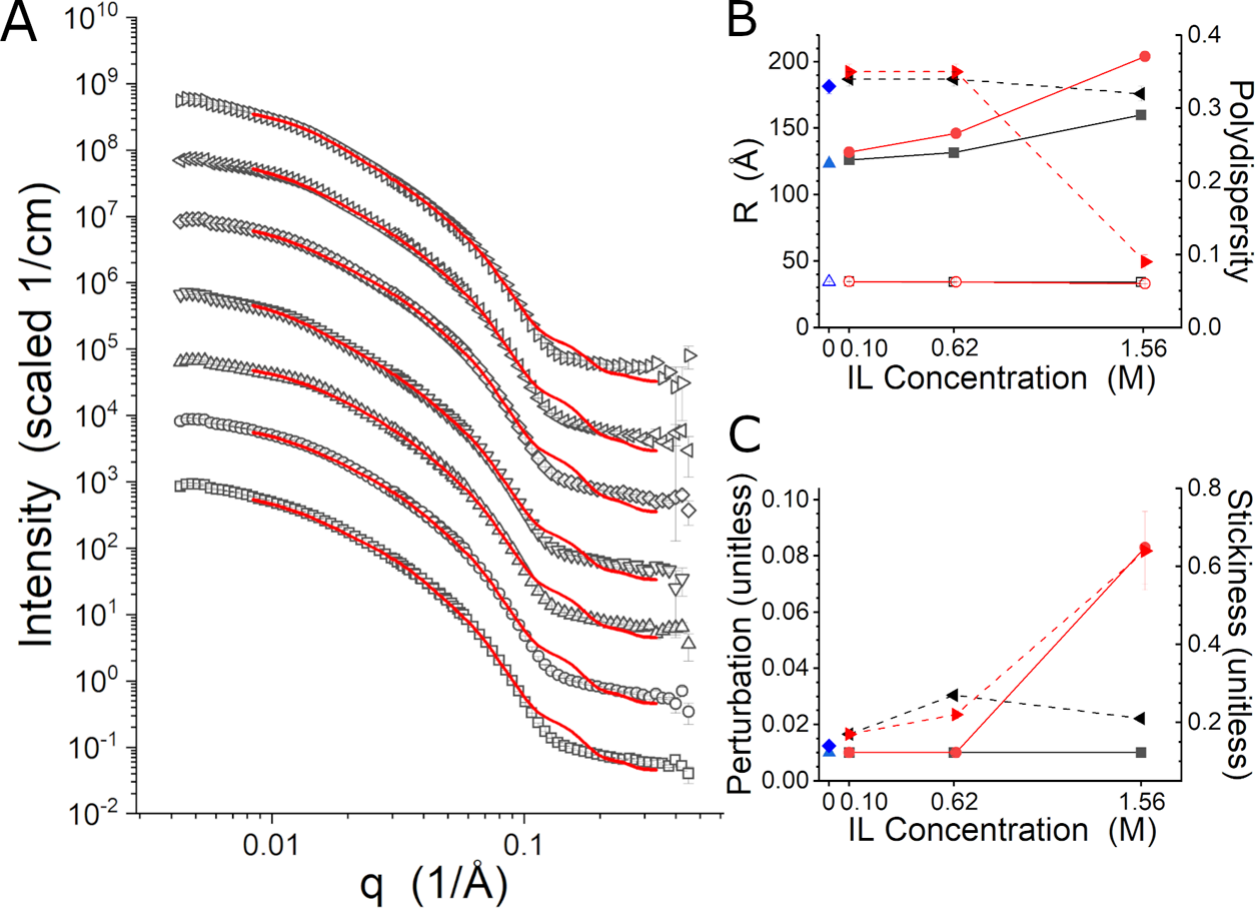
(A) SANS data identified as the large structure and the associated fit curves for no IL at 55 °C (squares), 0.10 M [C_4_C_1_im][Cl] at 55 °C (circles), 0.62 M [C_4_C_1_im][Cl] at 55 °C (up triangles), 1.55 M [C_4_C_1_im][Cl] at 55 °C (down triangles), 0.10 M [C_4_C_1_pyrr][Cl] at 55 °C (diamonds), 0.63 M [C_4_C_1_pyrr][Cl] at 55 °C (right triangles), and 1.57 M [C_4_C_1_pyrr][Cl] at 45 °C (left triangles). The red curves are the fit lines. The data and fit curves have been offset for clarity. (B) *R*_equat_ and *R*_polar_ for no IL (blue solid and open up triangles), [C_4_C_1_im][Cl] (black solid and open squares), and [C_4_C_1_pyrr][Cl] (red solid and open circles), along with the polydispersity in *R*_equat_, Δ_equat_ for no IL (blue diamonds), [C_4_C_1_im][Cl] (black left triangles), and [C_4_C_1_pyrr][Cl] (red right triangles). (C) “Perturbation” parameter from the sticky hard-sphere structure factor^46,51^ for no IL (blue up triangles), [C_4_C_1_im][Cl] (black squares), and [C_4_C_1_pyrr][Cl] (red circles), along with the “stickiness” parameter for the structure factor (blue diamonds), [C_4_C_1_im][Cl] (black left triangles), and [C_4_C_1_pyrr][Cl] (red right triangles).

The SANS data identified as the large structure were fit with an ellipsoid and a sticky hard-sphere structure factor.^53,58^ Previous studies of L62 have also used an ellipsoid to model SANS data collected in this temperature range,^43,46^ even though it is above the cloud point and therefore only partially descriptive of the system as a whole. However, it is useful for comparing the state of the system with the various concentrations of the ILs. The data analyzed were the sets collected at 55 °C, except for the 1.57 M [C_4_C_1_pyrr][Cl] sample. In this case, the 45 °C data were fit. The data and fit curves are shown in Figure 9A, although the high-*q* data are less well-fit than the data identified as the micelle state. The data and fitting suggest that the structure is less regular than an ellipsoid, which is evident for *q* > 0.10 Å^–1^. The deviations may be the result of the surface of the structure being considerably rougher than an ellipsoid. The structural parameters provided by the fitting are presented in Figure 9B,C. The *R*_polar_ for the structures found was consistent with the micelles presented in Figure 8B, but *R*_equat_ was consistently larger, as was observed previously for this Pluronic.^43,46^*R*_equat_ ranged from ∼120 Å for the salt-free sample up to ∼200 Å in the 1.57 M [C_4_C_1_pyrr][Cl]. It increased with increasing salt concentration, and the effect was stronger for [C_4_C_1_pyrr][Cl] than for [C_4_C_1_im][Cl]. The strength of the interparticle interaction generally increased when the ILs were present. This effect is visible in Figures 3 and 4 as the feature in the 0.01 Å^–1^ < *q* < 0.02 Å^–1^ range of the data sets with 10 vol % IL and higher. The stronger interaction also manifests in the perturbation and stickiness parameters in the results presented in Figure 9C, which are notably higher when the ILs are present at 0.62 M and higher. There is a clear trend of increasing interaction with increasing [C_4_C_1_pyrr][Cl] concentration but not with increasing [C_4_C_1_im][Cl] concentration.

## Discussion

The results presented here demonstrate that the ILs [C_4_C_1_im][Cl] and [C_4_C_1_pyrr][Cl] reduce the CP temperatures of the Pluronic studied. The morphologies adopted do not change, but the onset temperatures do. The [C_4_C_1_im]^+^ and [C_4_C_1_pyrr]^+^ cations impact the behavior of the polymer, rather than the effect depending entirely on the Cl^–^ anion. The ILs studied salt out the polymers and can be considered kosmotropes. [C_4_C_1_im]-based ILs have been extensively studied for a variety of applications, including biocatalysis. In particular, [C_4_C_1_im][Cl]-containing aqueous solutions denature proteins.^35−41,59−62^ The [C_4_C_1_im]^+^ cation was found to be a weaker kosmotrope than Na^+^.^36^ The impact of the ILs studied here is also consistent with the hydrotrope sodium xylene sulfonate.^48^ [C_4_C_1_pyrr]-based ILs are less studied than [C_4_C_1_im]-based ILs. However, the present results demonstrate that [C_4_C_1_pyrr]^+^ is a stronger kosmotrope than [C_4_C_1_im]^+^ for the L62 Pluronic.

The reduction of the CPs by the ILs studied here is consistent with other studies of the impact of salts on the solubility of Pluronics.^5−24^ The temperature- and salt concentration-dependent behaviors observed here are also consistent with how inorganic salts impact the self-assembly of Pluronics.^5−24^ SANS experiments showed that 1 M KCl lowers the CMT of F88 and causes the micelles to become larger in a manner analogous to how temperature increases micelle size.^7^ Similar behavior was seen for P65 with NaCl,^8^ P84 with KCl,^9^ L64 with NaCl,^14,19^ and F98 with NaCl.^22^ The relationship between temperature and salt concentration was not explored as extensively as it was in the present study, but the behavior seems consistent, suggesting that the pattern of self-assembly observed here is similar for inorganic salts. However, Fan and co-workers investigated the polymer series F38, F68, F88, F98, and F108, all of which have an 80% EO content, in aqueous solutions with 0.1 M Na_2_CO_3_ and found through SANS experiments that the salt induced a temperature-dependent effect on the structures formed that is very similar to the present results.^20^

The impact of the ILs on the structures found by SANS reveals interesting behavior. When they were present, the free L62 chains became somewhat more compact than when no salt was present, but there was no clear dependence of *R*_G_ on the IL concentration, but the presence of larger structures in the samples masked any differences. The micelles that were present were consistent in size if the lowering of the CMT and the other transition temperatures are considered. Like the micelles, the differences observed in the structure and interaction at temperatures above the CP are more likely the result of the temperature of the sample relative to the transition temperature into the state referred to here as the “highly interacting structure” state because of the strong correlation peak observed. Taken together, it is possible to conclude that the impact of the ILs on the structures formed by this Pluronic in aqueous solution is not great at a given temperature relative to a transition temperature, which is lowered by the IL in proportion to its concentration.

Some of the present results are consistent with previous studies of different Pluronics in aqueous solutions with a diverse array of ILs having different cations and anions.^63−76^ Generally speaking, ILs promote micellization, such as by decreasing the CMT, CMC, or CP, depending on the measurements that were made. Below, results from studies using ILs that were not similar enough in chemical structure to the present study, such as ethylammonium nitrate or cholinium-based ILs, will not be discussed in detail for the sake of brevity.^70,71,74,76^ Similarly, studies that focused on ILs more accurately characterized as surfactant-like (surface-active ILs or SAILs) due to the presence of an alkyl group having eight or more carbons will also not be discussed,^65,69^ unless ILs with short alkyl chains were included in the study.

In a study of [C_4_C_1_im][Br] with P104, the CMT was largely unchanged up to 1.2 M of the IL.^63^ Above this concentration, the CMT decreased. DLS revealed that the micelles grew with IL concentration when measured at a single temperature. The interaction of [C_5_C_1_im][BF_4_] with P123 was investigated by DLS and multiple spectroscopies.^64^ In this case, the micelles grew slightly at IL concentrations up to 0.3 M before decreasing in size as the concentration increased up to 0.9 M. A series of N-alkyl-pyridinium chlorides, [C_N_pyr][Cl] (*N* = 4, 6, and 8), at concentrations up to 0.2 M in the presence of P123 and F127 were studied by a variety of other techniques, including SANS.^67,68^ The presence of the ILs made the polymer micelles smaller, but the different alkyl chain lengths did not produce a clear trend for F127.^68^ In contrast, P123 micelles became more compact with both increasing IL concentration and alkyl chain length.^67^ The interaction of F108 with a series of [C_4_C_1_im]-based ILs with different anions was probed by DLS, UV–vis spectroscopy, NMR, and SEM. The anions investigated were SCN^–^, BF_4_^–^, I^–^, Cl^–^, C_2_H_3_O^–^, and HSO_4_^–^ at concentrations up to 15 mg/mL, which is less than 100 mM for all of the ILs studied.^66^ DLS revealed the growth of the micelles over the temperature range of 30–45 °C, and the positive or negative impact of the anion on the onset of the growth follows the Hofmeister series for ILs.^35,36^ The effect was also stronger for kosmotropes than for chaotropes. F127 was also studied by DLS in aqueous solutions containing [C_N_C_1_im] halide ILs (*N* = 4, 6, and 8) at 100 mM.^72^ The study found that the micelles became smaller with the various ILs. However, there was no strong trend related to the alkyl chain length. Similarly, F108 in aqueous solutions of [C_N_C_1_im][Cl] ILs (*N* = 2, 4, 6, and 10) at IL concentrations under 100 mM was investigated through a combination of DLS and spectroscopies.^73^ The hydrodynamic radius of the micelles increased with increasing IL alkyl chain length, and the CMT decreased and the hydrodynamic radius of the micelle increased relative to that of the IL-free micelle at a fixed temperature, but the size of the micelle was similar at a temperature a fixed difference above the CMT.

The picture painted about the impact of ILs on the self-assembly of Pluronics by the current and previous works is complex,^63,64,66−68,72,73^ owing mostly to the diversity of the Pluronics and ILs studied, but there is some consistency. Other researchers found that ILs change the size of the micelles formed at a given temperature and the cation and anion effects generally follow Hofmeister trends.^35−37^ The impact of the ILs on the temperature-dependent behavior of L62 is different from a study of several [C_N_C_1_im]^+^-based ILs, including [C_N_C_1_im][Cl], and the F108 Pluronic that found larger micelles formed at a temperature relative to the CMT.^66^ F108 has a great deal more (EO) groups per chain and relative to the number of (PO) groups in a single chain. It is reasonable to also expect that some of the different behaviors seen are the results of the different ratios of EO to PO in the various Pluronics studied. When put in the context of previous work, the present results suggest that the total amount of (EO) groups in a chain is an important determinant of how the interaction of a Pluronic with an IL impacts the self-assembly of the Pluronic.

## Conclusions

The temperature- and IL concentration-dependent behaviors of aqueous solutions of the Pluronic L62 were investigated by SANS. The ILs change the temperature dependence of the self-assembly of the polymer. Both [C_4_C_1_im][Cl] and [C_4_C_1_pyrr][Cl] lower the CMT of L62 in a manner like inorganic salts and other ILs. [C_4_C_1_pyrr][Cl] has a stronger effect on the CMT than [C_4_C_1_im][Cl]. However, neither IL has a large impact on the structures formed at a constant temperature relative to the temperatures at which structural transitions take place. The new insight gained into the relationship between IL concentration and temperature dependence of the self-assembly of L62 demonstrates how the self-assembly of this Pluronic in water can be tuned using ILs having different cations without altering the various structures that it forms.
